# Supplementary figures and images for: Optimization of nutritional strategies using a mechanistic computational model in prediabetes: Application to the J-DOIT1 study data
Source: PLoS One. 2023 Nov 30;18(11):e0287069. doi: 10.1371/journal.pone.0287069 (PMC10688723; doi:10.1371/journal.pone.0287069)

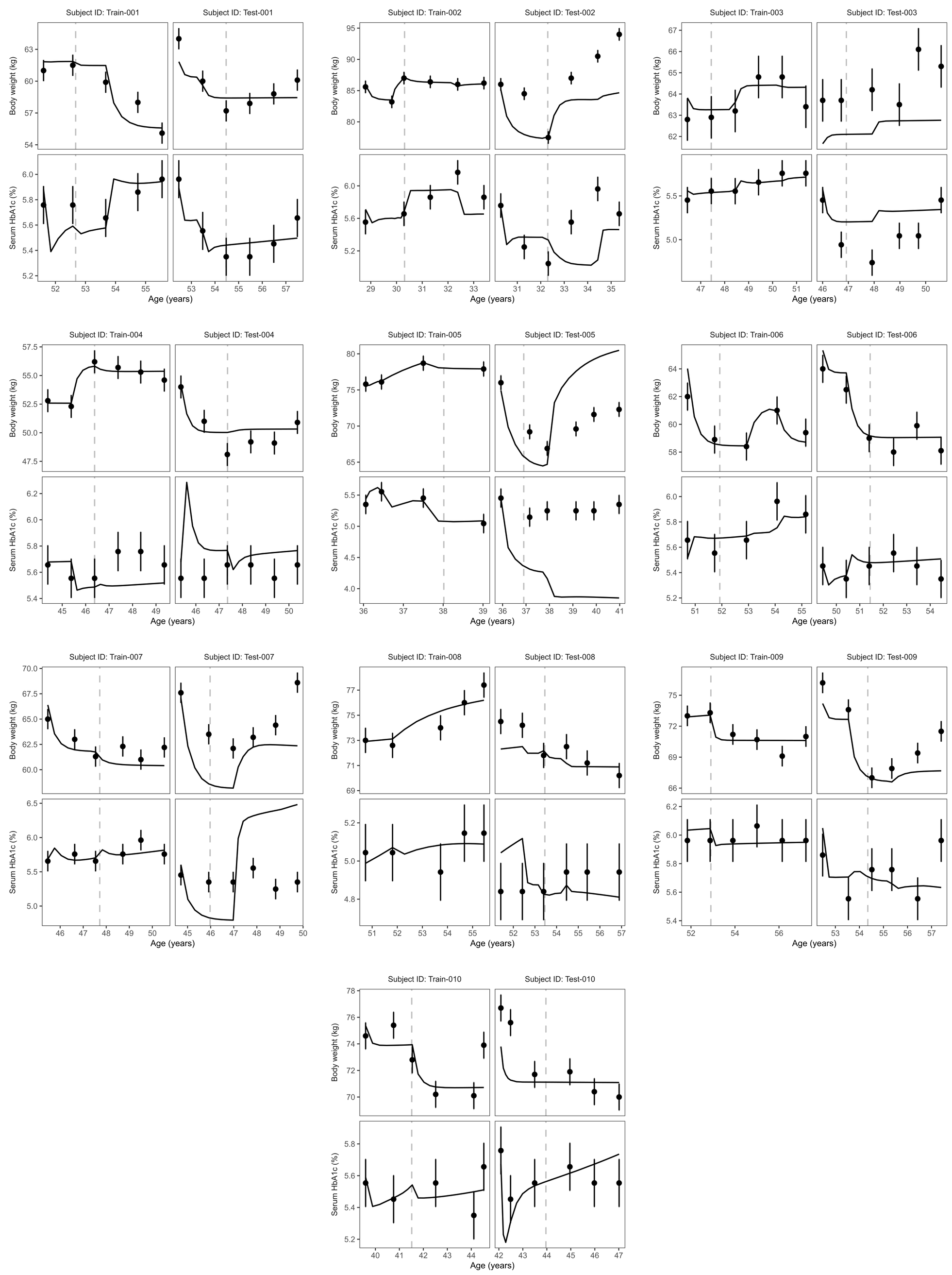

Supplement: S1 Fig — (TIF) [file pone.0287069.s001.tif]

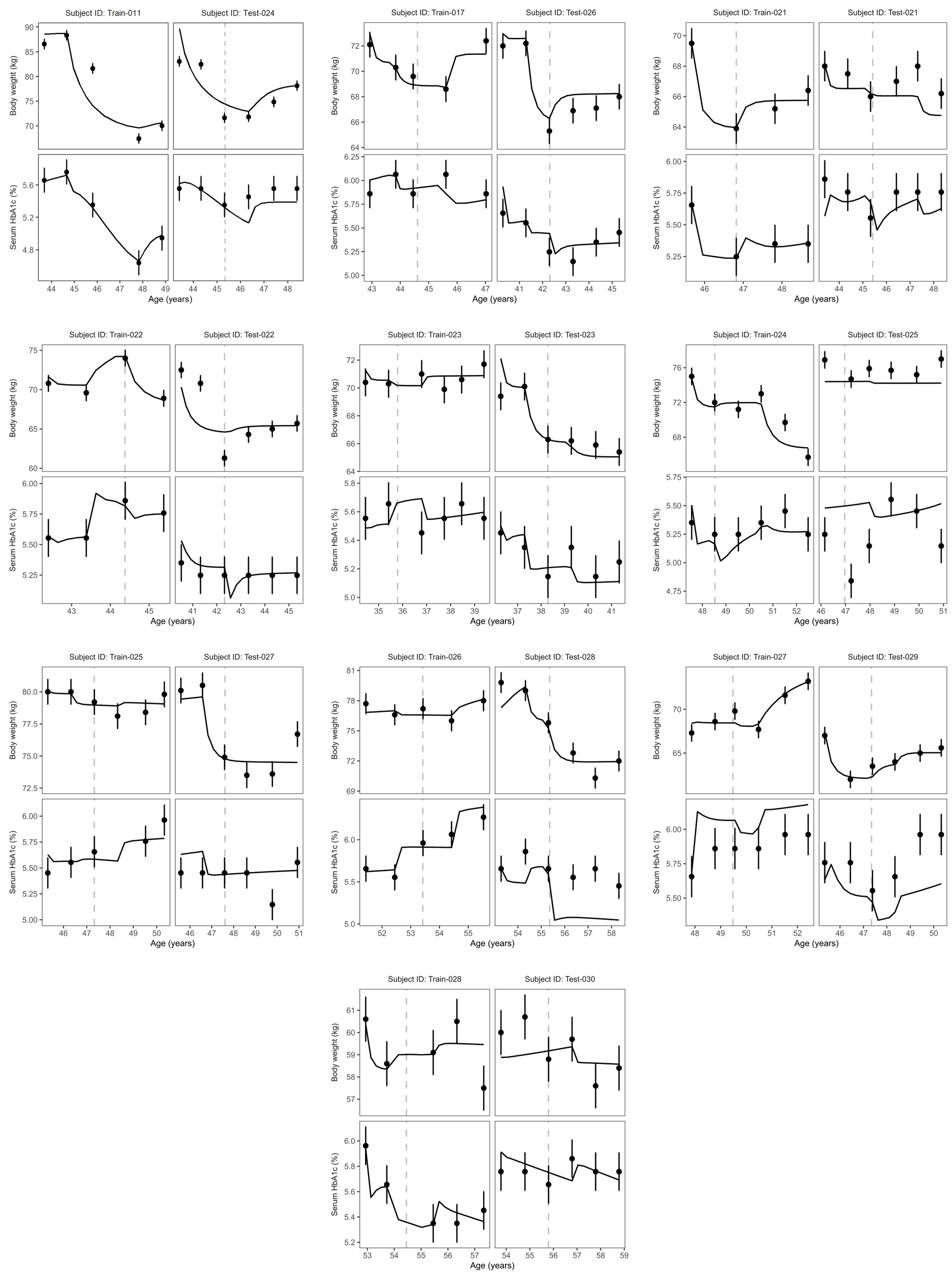

Supplement: S2 Fig — (TIF) [file pone.0287069.s002.tif]

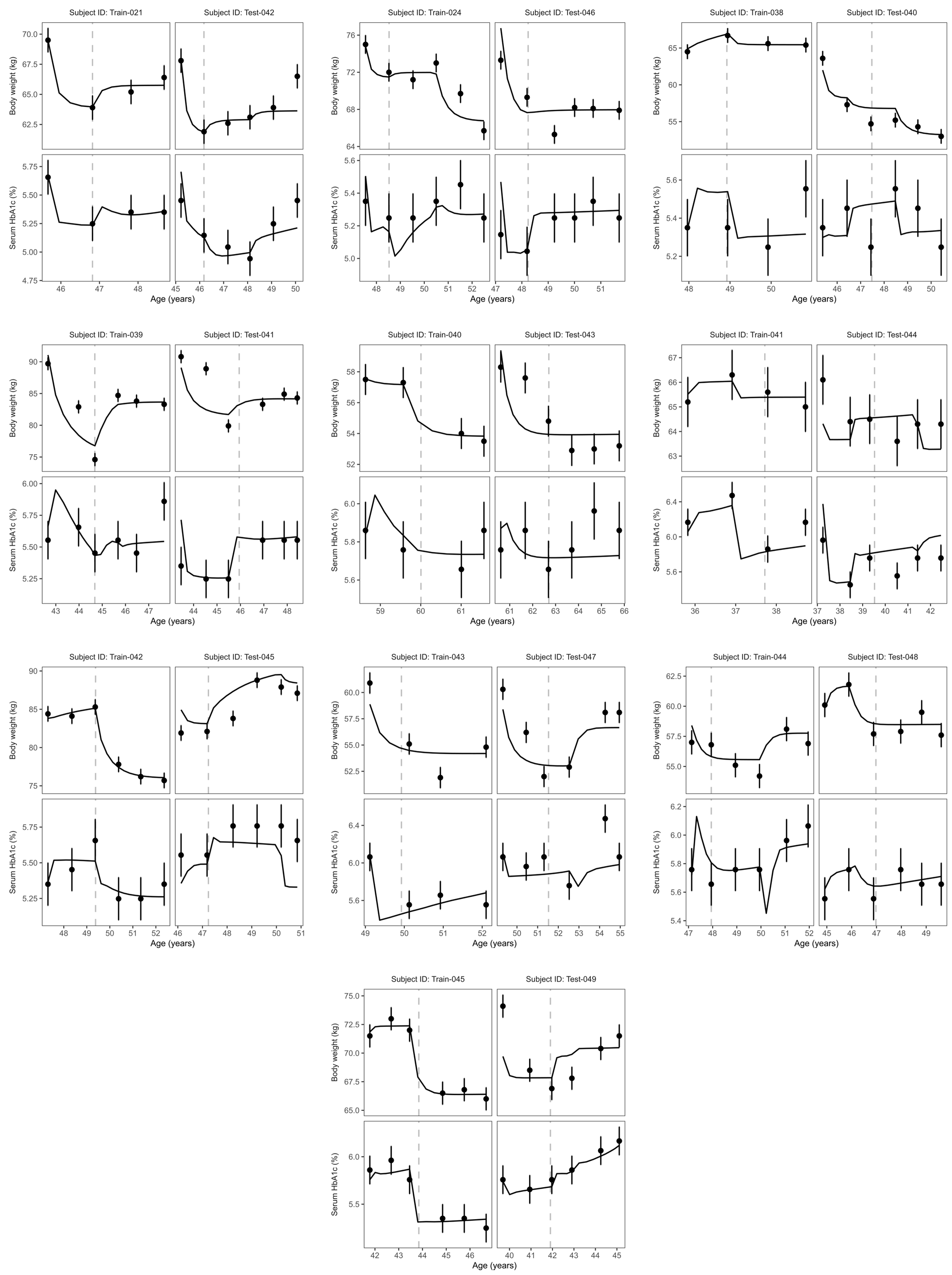

Supplement: S3 Fig — (TIF) [file pone.0287069.s003.tif]

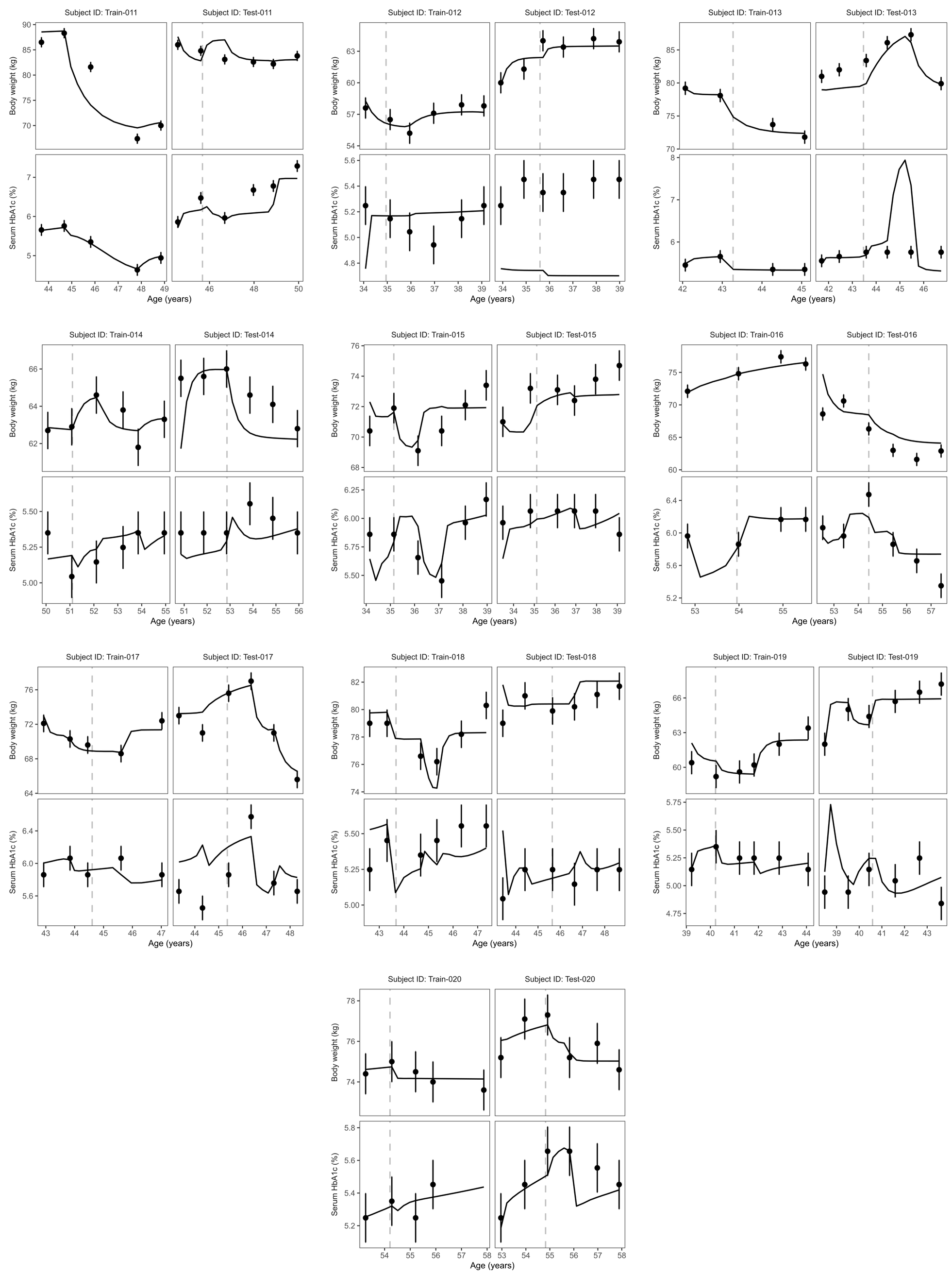

Supplement: S4 Fig — (TIF) [file pone.0287069.s004.tif]

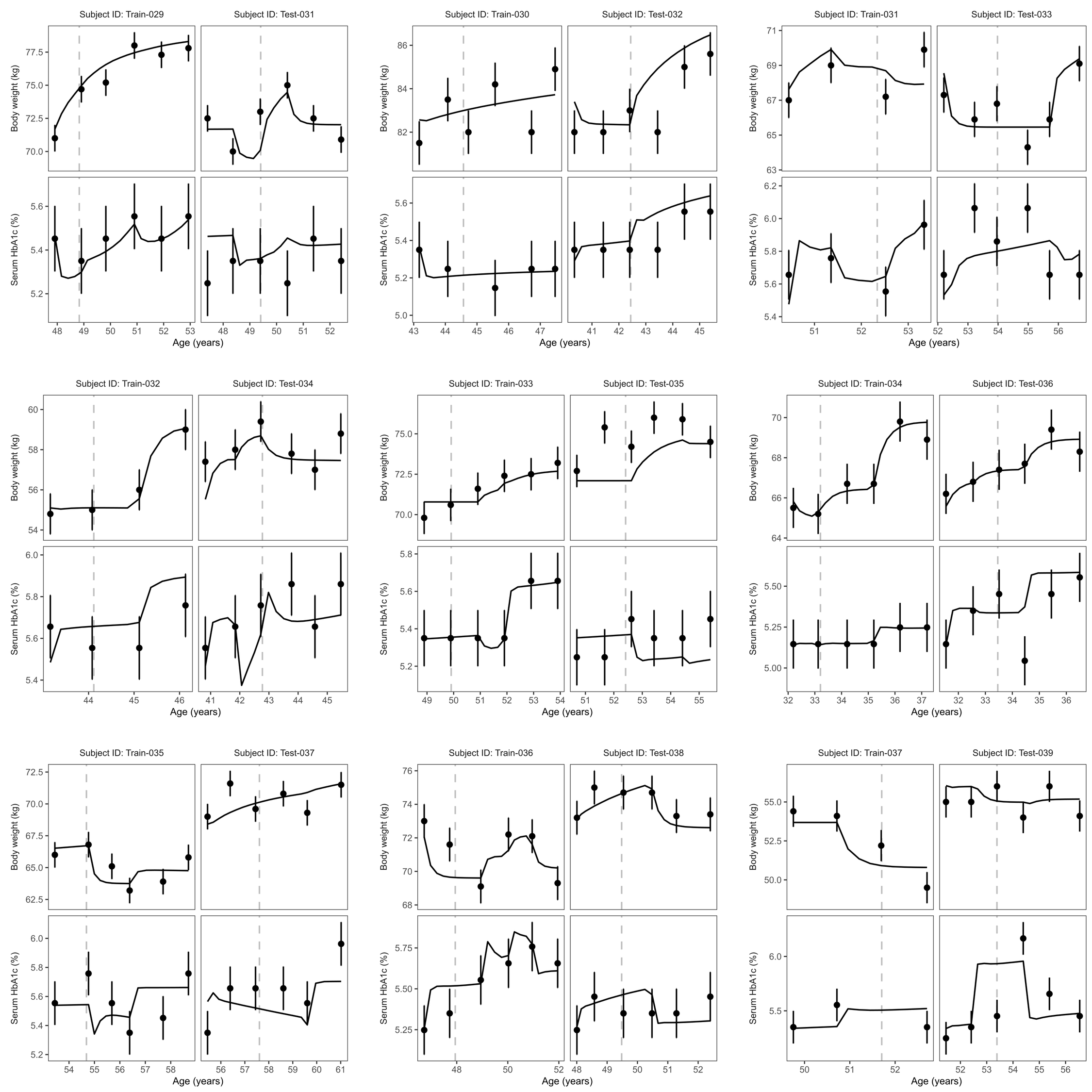

Supplement: S5 Fig — (TIF) [file pone.0287069.s005.tif]

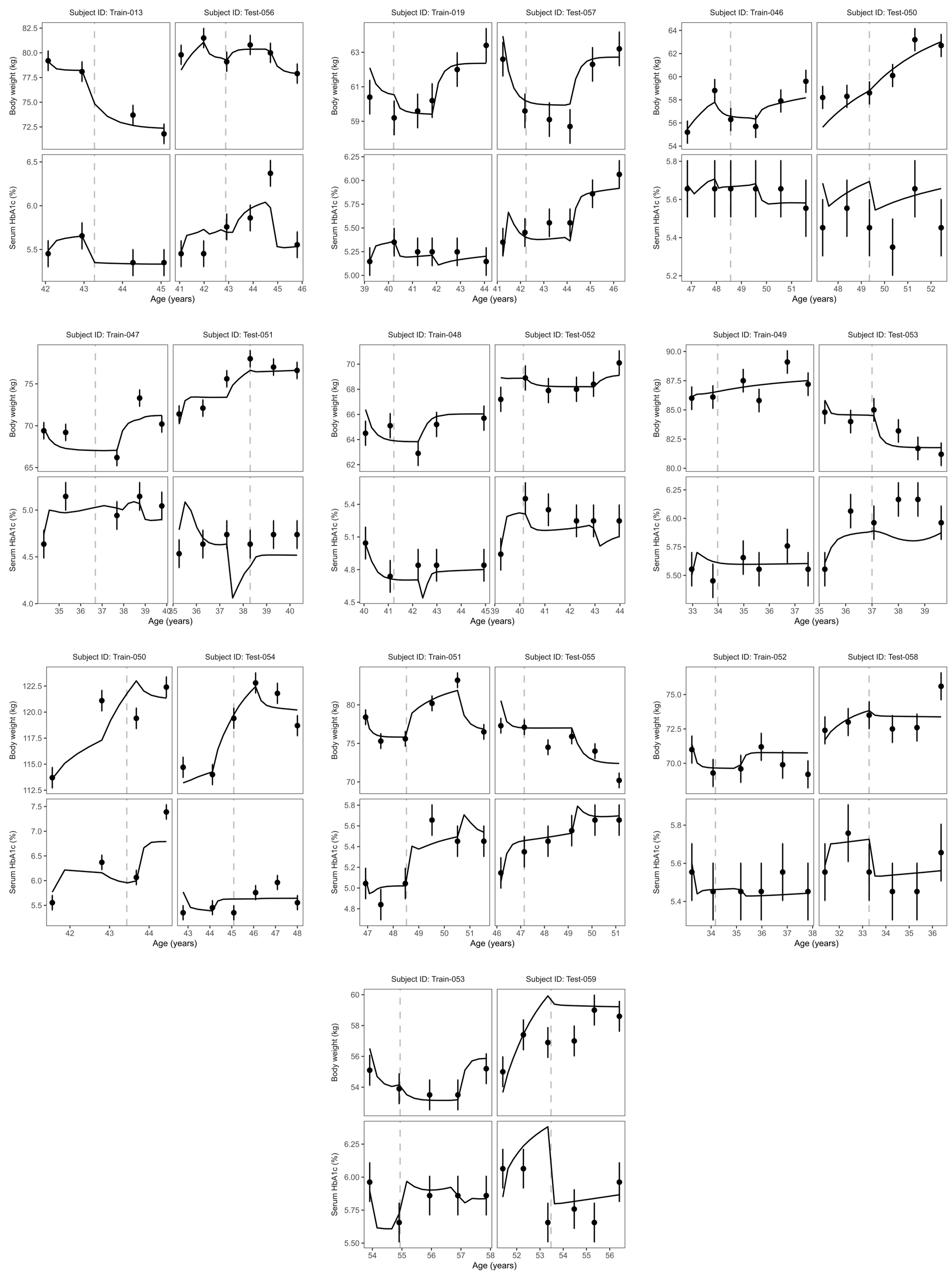

Supplement: S6 Fig — (TIF) [file pone.0287069.s006.tif]

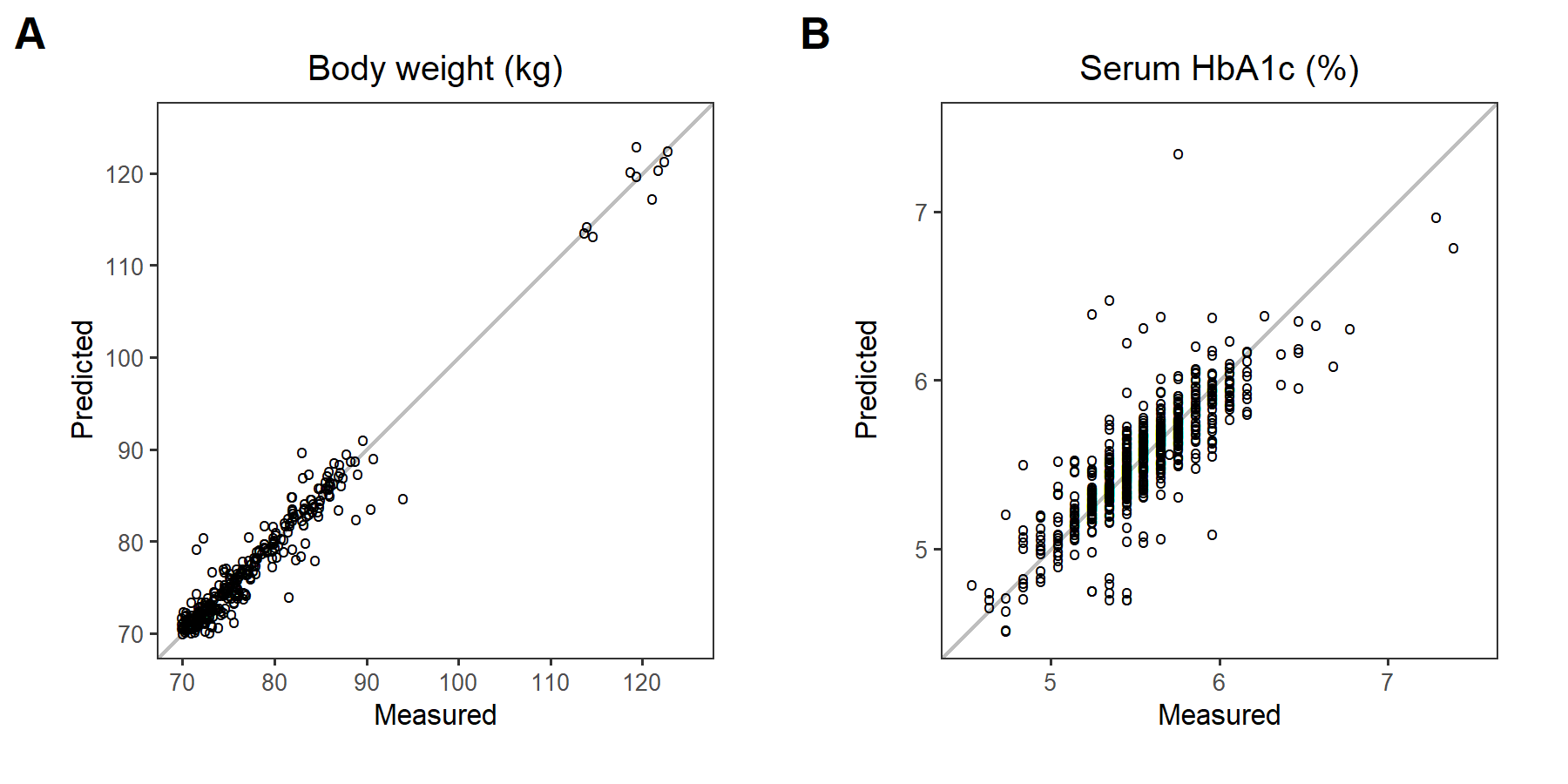

Supplement: S7 Fig — Model-predicted body weight and HbA1c values for all subjects across time points show reasonable concordance with corresponding measured values with most values lying on or close to the line of identity. (TIF) [file pone.0287069.s007.tif]

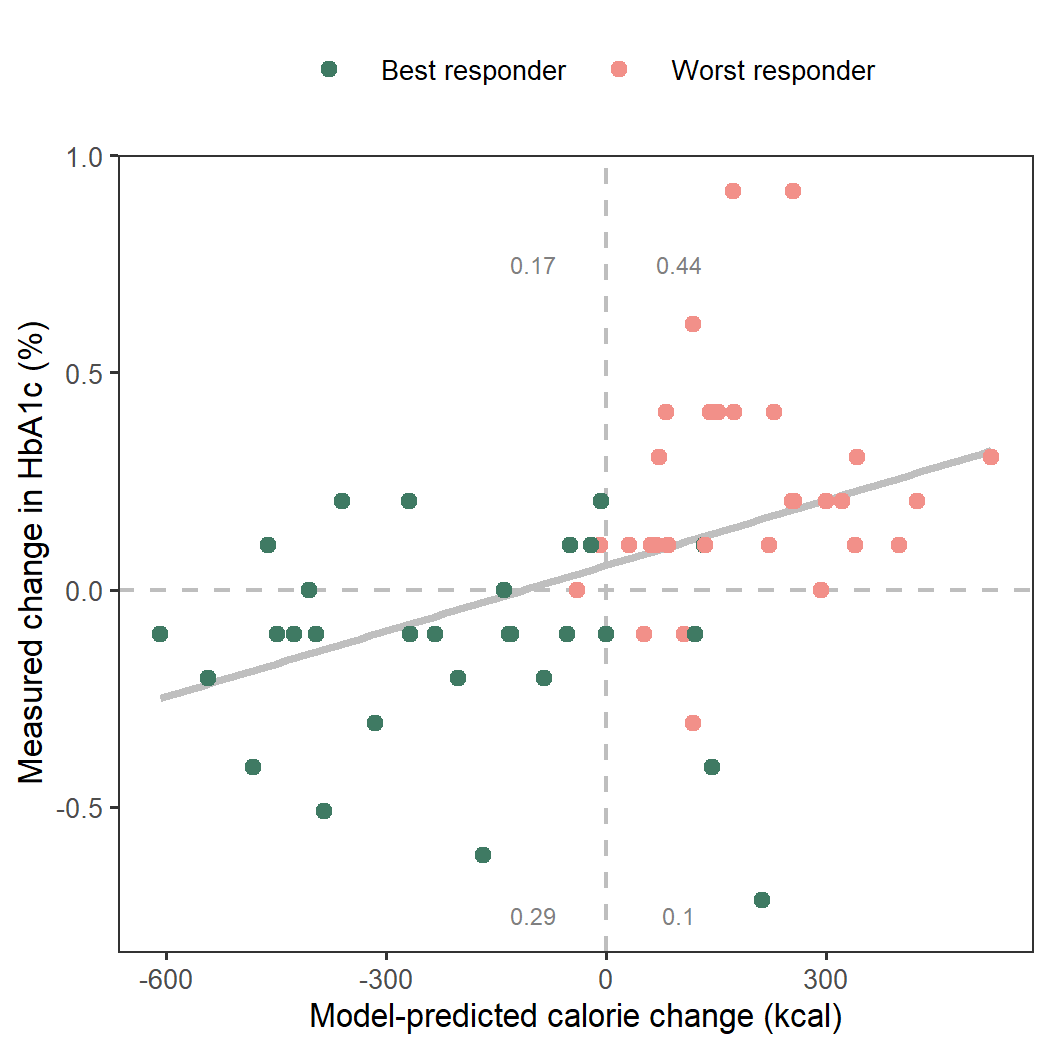

Supplement: S8 Fig — The measured change in HbA1c from baseline to the first follow-up during the J-DOIT1 intervention plotted against model-estimated change in calories per day due to both diet and exercise changes averaged over the same period for subjects in the intervention arm. The gray number in each quadrant is the fraction of data points in that quadrant. The data points fit a linear regression model (solid gray line) with r2 = 0.20 and a residual standard error of 0.28 points. (TIF) [file pone.0287069.s008.tif]

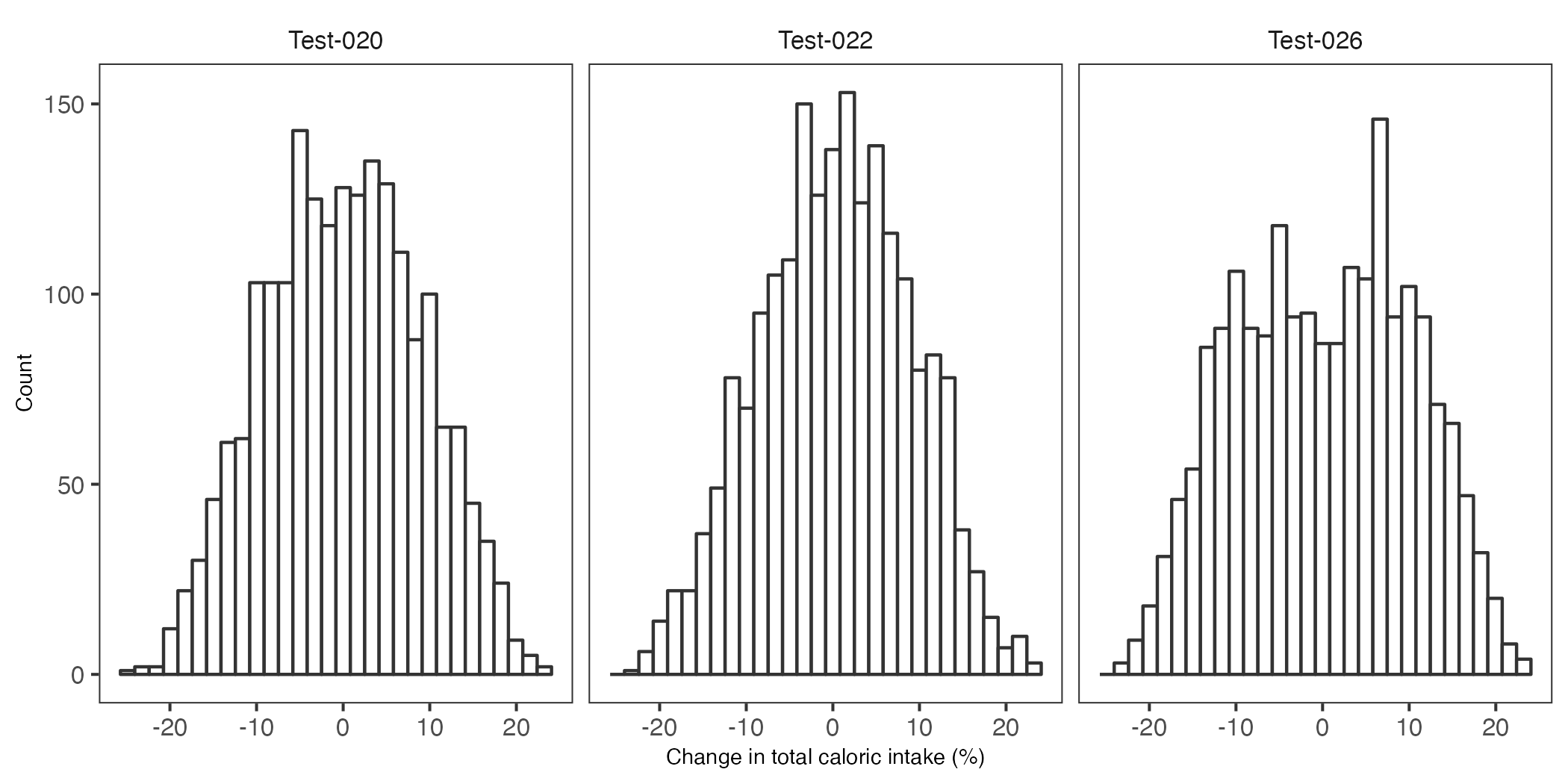

Supplement: S9 Fig — Total caloric changes sampled to find optimal diets are shown for three randomly selected subjects (Test-020, Test-022, and Test 026). Total caloric change is approximately normally distributed with mean 0 and covers the ±25% range. (TIF) [file pone.0287069.s009.tif]

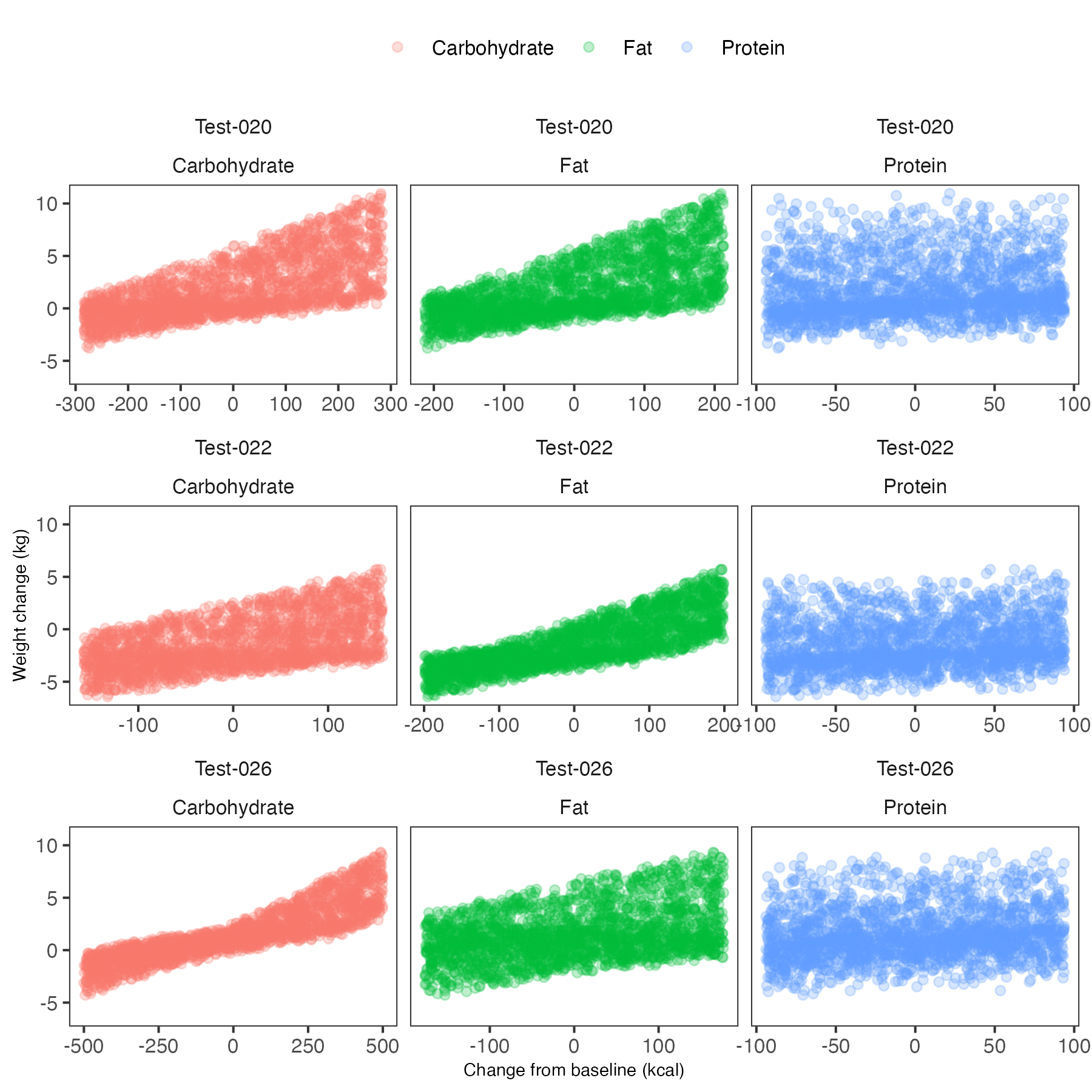

Supplement: S10 Fig — All sampled macronutrient changes (N = 2000 per subject) were plotted against the model predicted weight change for three randomly selected subjects (same subjects as in S9 Fig). Carbohydrate and fat changes are, on average, monotonically related with weight change; however, protein changes are not correlated with weight change. (TIF) [file pone.0287069.s010.tif]
